# Bad News: Analysis of the Quality of Information on Influenza Prevention Returned by Google in English and Italian

**DOI:** 10.3389/fimmu.2015.00616

**Published:** 2015-12-08

**Authors:** Ali Maki, Roger Evans, Pietro Ghezzi

**Affiliations:** ^1^Clinical and Laboratory Investigations, Brighton and Sussex Medical School, Falmer, UK; ^2^School of Computing, Engineering and Mathematics, University of Brighton, Moulsecoomb, UK

**Keywords:** internet, information quality, Google, websites, influenza, vaccine, prevention, information

## Abstract

Information available to the public influences the approach of the population toward vaccination against influenza compared with other preventative approaches. In this study, we have analyzed the first 200 websites returned by searching Google on two topics (prevention of influenza and influenza vaccine), in English and Italian. For all the four searches above, websites were classified according to their typology (government, commercial, professional, portals, etc.) and for their trustworthiness as defined by the Journal of the American Medical Association (JAMA) score, which assesses whether they provide some basic elements of information quality (IQ): authorship, currency, disclosure, and references. The type of information described was also assessed to add another dimension of IQ. Websites on influenza prevention were classified according to the type of preventative approach mentioned (vaccine, lifestyle, hygiene, complementary medicine, etc.), whether the approaches were in agreement with evidence-based medicine (EBM) or not. Websites on influenza vaccination were classified as pro- or anti-vaccine, or neutral. The great majority of websites described EBM approaches to influenza prevention and had a pro-vaccine orientation. Government websites mainly pointed at EBM preventative approaches and had a pro-vaccine orientation, while there was a higher proportion of commercial websites among those which promote non-EBM approaches. Although the JAMA score was lower in commercial websites, it did not correlate with the preventative approaches suggested or the orientation toward vaccines. For each of the four search engine result pages (SERP), only one website displayed the health-of-the-net (HON) seal. In the SERP on vaccines, journalistic websites were the most abundant category and ranked higher than average in both languages. Analysis using natural language processing showed that journalistic websites were mostly reporting news about two specific topics (different in the two languages). While the ranking by Google favors EBM approaches and, in English, does not promote commercial websites, in both languages it gives a great advantage to news. Thus, the type of news published during the influenza season probably has a key importance in orienting the public opinion due to its high visibility. This raises important questions on the relationships between health IQ, trustworthiness, and newsworthiness.

## Introduction

### Prevention of Influenza

Influenza is a highly contagious viral respiratory disease with a significant burden on public health (1, 2). Influenza causes symptoms of fever, muscle pain, headache, cough, and, in more vulnerable patients, it can cause exacerbations of respiratory disease, which can sometimes be life threatening (1). There are not many effective treatment options available, so interventions focus on prevention, with a number of interventions identified by the Cochrane Library to have a sufficient enough evidence base (1–4).

Vaccination is the most recommended preventative measure. Unlike other vaccines, the influenza vaccine provides a modest protection for adults, with children over the age of two showing far better benefits than adults (1, 2) and elderly people showing less benefit (1). Most government agencies often recommend vaccination only in populations at risk of developing serious complication from influenza, such as the over 65 age group, people who are immunosuppressed, those who have underlying health conditions such as cardiovascular or respiratory diseases, pregnant women, and healthcare workers.

Hand washing is also effective in preventing the spread of influenza; this was again particularly true for children (3). Surgical masks as physical barriers were also found to be an effective preventative intervention (3). Antiviral drugs such as Tamiflu can have a prophylactic effect (4, 5), although possible side effects and the obligation to not medicate unnecessarily make this intervention something that should only be considered by those at high risk of infection.

Vitamin C supplementation is also popular as a preventative treatment for influenza, based on publications by Linus Pauling in the 70s (6). A Cochrane review on vitamin C in the prevention of common cold, which is caused by respiratory viruses including influenza, could not find evidence of a reduced incidence of the disease although there was a reduction in its duration (7). There is also no evidence for a preventative effect on pneumonia, another complication of influenza (8).

Other interventions such as certain homeopathic remedies and increased fluid intake showed no evidence of preventative benefits (9, 10).

### Quality of Health Information and Impact on Public Health

Statistics show that up to 59% of the total adult population has at some point searched for health information online, with this proportion set to continually rise (11). Patients can become quite reliant on this ease of access, choosing to refer to the Internet whenever they have a query, with a large number eventually making a decision based on their search (12). This is further helped by the anonymous nature of the Internet, which provides a certain degree of confidentiality to the patient. Thus, the Internet has allowed patients to take a more dynamic role when seeking health information and maintaining their own health (11).

While the benefits of the Internet are clear, there are numerous concerns regarding the quality of information found online and the reliability of sources available. The Internet remains a largely unregulated entity, and while tightly regulated websites do exist, any individual can set up a website and broadcast information that is potentially unreliable or inaccurate, which may harm patients or professionals who choose to act on that information (12, 13).

The information that can be retrieved using search engines is varied and it is not just made of “standalone” websites. In fact, most of the websites in a typical search engine result page (SERP) are not independent sources of information, but are owned by magazines, newspapers, or TV news channels, and just reproduce what is reported by other types or media. A significant proportion of websites are government health agencies, patient advocacy groups, or pharmaceutical companies, posting on their websites information that is also available in their printed brochures. What is important in the use of a search engine is the proportion of these different types of websites returned in the SERP as well as their ranking, because most users will often look only at the first websites in the list (14, 15).

### Assessing Health IQ

The aim of this study was to assess the quality of the information returned by Google on the prevention of influenza in English and in Italian.

Information quality (IQ) has several dimensions (16, 17). Some of them, such as accessibility or readability (e.g., it is written in a clear language or can be accessed without a paywall), are difficult to transfer some of these concept to health information. We analyzed two dimension of IQ that seems more directly relevant. The first is the trustworthiness as defined by the Journal of the American Medical Association (JAMA) benchmarks, a tool to help users seeking to evaluate the reliability of a website (18). The benchmark consists of four criteria: authorship, attribution (referenced sources), currency (articles dated), and ownership disclosure (including financial interests). Websites that fulfill three or more of these criteria are deemed reliable and more likely to contain higher quality information (18). The tool has since become a widely established method for assessing quality when evaluating health information online. The second dimension is accuracy. Accuracy has been described when “the recorded value is in conformity with the actual value” (19), and is therefore equivalent to correctness (16). In our context, this could be interpreted as “scientifically sound” or “evidence-based.” For instance, a website describing influenza as an infection by a virus would be accurate whereas one describing it as a disease due to malnutrition, rather than a virus, would be inaccurate.

One approach used to assess scientific accuracy is to have websites scored by medically trained reviewers (20). Evidence-based medicine (21) is now widely used by regulatory agencies to approve new drugs and as a rational approach in the identification of effective treatments to be reimbursed by health insurance systems or offered by public health services. In a previous study, using websites on migraine as an example, we have used as an indicator of scientific accuracy whether or not websites were pointing the reader toward treatment options that were evidence based (22).

In this study, we have followed a similar methodology, classifying the type of websites returned by a SERP on “preventing the flu” in terms of class of website (commercial, government agencies, news, etc.) and type of intervention described (vaccine, approved drugs, complementary-alternative medicine, nutritional approach, lifestyle, hygiene). The websites were also scored for each of the four components of the JAMA criteria (authorship, attribution, currency, disclosure). We searched Google for the expression “preventing flu” (the layperson expression for influenza in English) or the Italian equivalent “prevenzione influenza.” We analyzed the first 200 websites listed in the SERP to obtain a representative sample of the information available on the web. Because most people will only look at the first 10 websites or less (14, 15), we also performed a subgroup analysis on the top 10 results in the Google SERP.

Finally, we extended the study to the information on influenza vaccine, analyzing the first 200 websites returned by a search on “flu jab” (English) or “vaccino antinfluenzale” (Italian) and scoring them as above except that, instead of intervention type, we classified them according to their stance on vaccination: pro, anti, or neutral.

We then performed a hierarchical cluster analysis to identify website patterns and used natural language processing (NLP) software to identify key new topics.

The results of the analysis illustrate the different behavior of the differences in IQ across the different classes of websites and the impact of the news on the health information found through Google.

## Materials and Methods

A search was conducted in December 2014 for information relating to the prevention of influenza infection using the Mozilla Firefox web browser and the first 200 search results were downloaded onto a spreadsheet using the SEOquake software toolset (Semrush Inc., Trevose, PA, USA).

The Google^1^ search engine was chosen due to its popularity among web users. The search was carried out using the “private mode” function of the browser, as this prevented the possible interference of past searches on current results, although we are aware that the IP address revealed our (UK) location within the University campus and that may influence the results obtained. The phrase “preventing the flu” was selected as the search input as it was deemed an appropriate phrase likely to be used by the general public in British English. In fact, it has been shown that using technical language is more likely to return results of higher quality (23, 24). The search in Italian was done on Google, limiting the language of the results to Italian and using the search term “vaccino antinfluenzale.” We did not take into consideration the websites labeled as “Google ads” that appear at the top of the SERP.

The websites were then visited individually by two researchers and classified as described below. Whenever a website was classified differently by the two researchers, the website was revisited and discussed to reach a consensus. Websites that were not accessible since they were downloaded or that were accessible only via a paywall were excluded. Irrelevant websites (e.g., on swine flu, advertisement for conferences, polls, off topic) were also excluded. The spreadsheet with the list of websites and the way they were classified is available in Tables S1–S4 in Supplementary Material, to allow reader to reanalyze or reclassify according to different criteria.

### Classification of Websites

**Figure 1:**
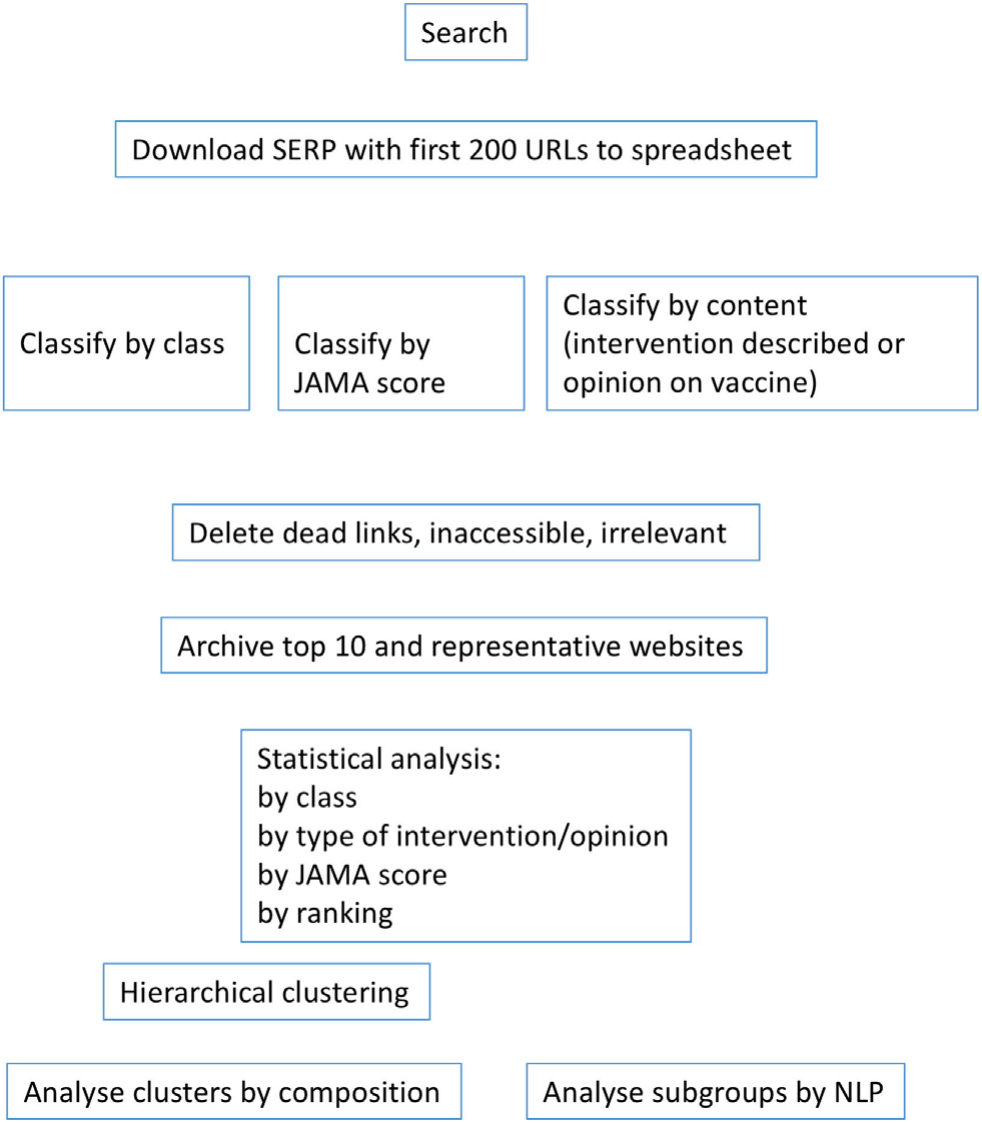
**Workflow for the analysis of websites**.

The workflow for the study is described in Figure 1. Websites were classified in accordance to their affiliation as government, commercial, journalistic, professional, portals, non-profit organizations, or others as indicated in Table 1. Although in previous studies, we did not classify governmental website separately, we felt it was useful to do so here because of the large number of such websites returned by Google for this topic. The inter-rater agreement between the two markers was 96% for the search on flu prevention in English, 90% for the search on the vaccine in English, 97% for prevention in Italian and 98% for the vaccine in Italian.

**Table 1:** **Website categories, descriptions and examples**.

Purpose	Description	Example
Government (G)	A website created, managed, and regulated by an official governmental body	cdc.gov
		flu.gov
		nhs.uk
Commercial (C)	A website specifically created for commercial purposes and the sale of products or services with the aim of making a profit	tamiflu.com
		myprotein.com
Journalistic (J)	A website created for the distribution of news and information. Covers general news mediums as well as health related ones. Can also be for entertainment purposes	cnn.com
		theguardian.com
		menshealth.com
Professional (P)	A websites created by health professionals, experts, and professional organizations	mayoclinic.com
		thecochranelibrary.com
Health Portal (HP)	A webpage with a search function that accesses information and articles from the site for a range of health topics	webmd.com
		mcmasteroptimalaging.org
Non-profit Organization (NPO)	An organization with charitable/supportive/educational services that are not established for the purpose of profit	redcross.org
		bhf.org.uk
Other (O)	Websites that do not fit the criteria for other categories, such as personal blogs and social networking sites	twitter.com
		wikihow.com

Websites on prevention of influenza, in both languages, were then analyzed, and any interventions recommended were recorded. These recommendations were assigned to one of the following “types of intervention” similarly to our previously study (22): vaccine (also known as “Flu jab”), approved drug, lifestyle, hygiene, supplements, nutrition, complementary and alternative medicine (CAM) herbal products, CAM practices, and devices. Only when an intervention was mentioned in a neutral or positive way the website was classified in that particular group (i.e., an anti-vaccine website that mentioned the vaccine only to recommend not to use it would not be classified as “vaccine”). Table 2 details the criteria used for the classification.

**Table 2:** **Descriptions of types of preventative intervention**.

Intervention	Examples
**Flu vaccine**	
Approved Drug	A pharmaceutical therapy that has been clinically approved by a regulatory agency (e.g., FDA, EMA, MHRA) for use in preventing influenza
Lifestyle	Lifestyle factors and changes. E.g., exercise, staying warm, and good sleep patterns
Hygiene	Washing hands, keeping distance from infected individuals
Supplements	Specific dietary additions that are usually taken in higher concentrations than what is normally found in food. E.g., vitamins, antioxidants, metals
Nutrition	Foods and meals
CAM Herbs	Herbal substances (e.g., Echinacea)
CAM Practices	Alternative medicine practices such as massages
Devices	E.g., facemasks

Websites returned from the two specific searches on the vaccine (English and Italian) were also classified as positive (recommending the vaccination or reporting a recommendation), negative (recommending not to use the vaccine or specific anti-vaccine sites), or neutral (just reporting the opening hours to have a vaccination, news reporting of the efficacy/weakness of the vaccine or incidents attributed to vaccination).

Finally, each website was assigned a JAMA score as described previously (22, 25). Briefly, a score of 1 was assigned for each of the following four informations present in the webpage: (1) authorship (name of the author of the text); (2) attribution (references provided to back up statements); (3) currency (indication of the date of publication and/or update); and (4) disclosure (website describes ownership and commercial interests). These scores where then added up giving a JAMA score between 0 and 4.

If the information was not available on the initial website page, then the three-click rule was used. The three-click rule is an unofficial website navigation rule that suggests information should be accessible within three clicks (26) In previous studies, a website scoring a mean JAMA score of 3 or above has been suggested to be of high quality (27, 28).

### Statistical Analysis

The Kruskal–Wallis test was used for multiple comparisons of non-parametric variables, followed by Dunn’s test, using GraphPad Prism software (GraphPad Prism Software Inc., La Jolla, USA). The Mann–Whitney test was used where there were two independent groups. When indicated, contingency tables were analyzed using a Chi-square test for non-parametric data. Hierarchical cluster analysis was performed using the Genesis software (University of Graz, Austria; version 1.7.6 for Mac OSX).

Because websites URLs are not permanent, to ensure that the reader will be able to see examples of the search results, the top 20 URLs returned in each of the Google SERPs as well as examples of some types of websites were archived. For this purpose, we used WebCite^®^, an on-demand archiving system for web references and the archived URL is provided next to the original URL. For webpages that could not be archived, either because the link to the site in question was already dead or the website refused connections by crawling robots, the archive link is indicated as not available (n/a).

### Natural Language Processing

Four corpora were created from the lists of URLs in each SERP using WebBootCaT, an online tool for bootstrapping text corpora from Internet, and analysis of natural language was performed using the corpus analysis software Sketch Engine^2^ (29). Bi-gram and tri-gram (repeated 2 and 3 word sequences in the text) frequency lists were compiled for each corpus, with obvious words (vaccine or influenza and their derivate in both languages, and common words, also known as “stop” words) not considered.

## Results

### Searching Google for Influenza Prevention

**Figure 2:**
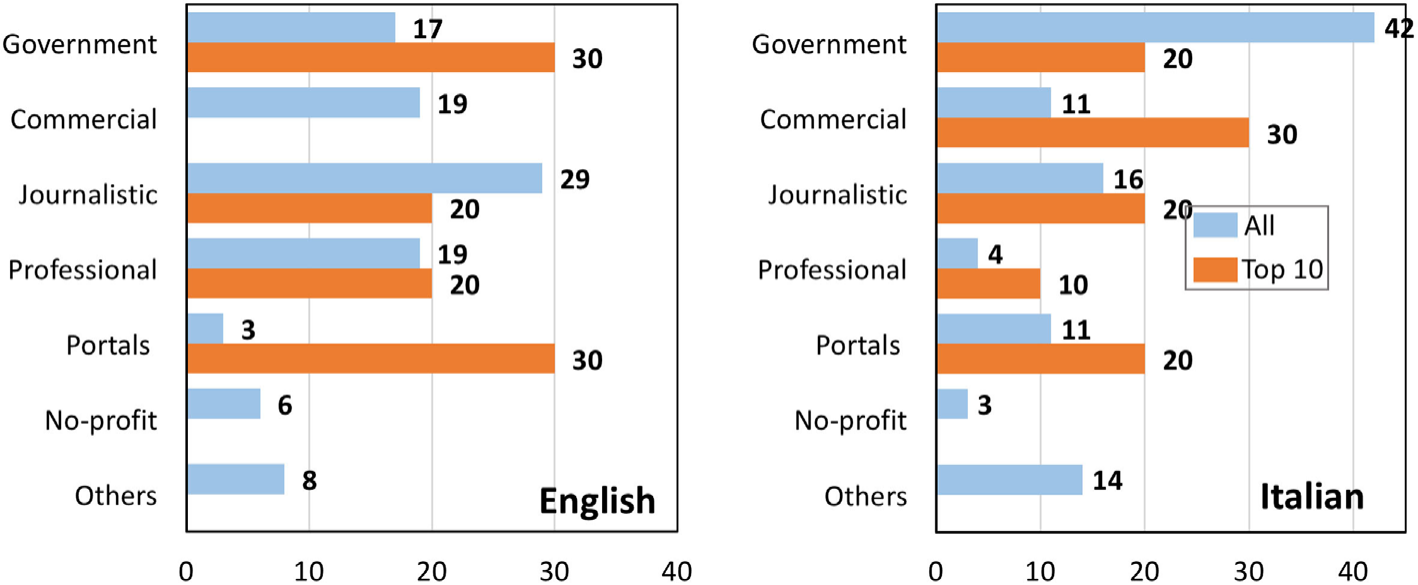
**Percentage of different classes of websites from a search on influenza prevention in English (left) or Italian (right)**. Blue bars, all websites; orange bars, top 10 results returned by Google.

Figure 2 shows the types of websites returned by Google when searching for influenza prevention in English or Italian. For each search, we analyzed the overall SERP (blue bars) or the top 10 websites only (orange bars). For the search on prevention in English, the most represented class of websites were journalistic (29%), followed by professional, commercial, and government (all around 18%). However, if we look at the top 10 websites in both searches, there are no commercial websites and government websites are prevalent, along with portals (each being 30% in top-10). It should be noted that the high percentage of portals is due to the presence of three www.webmd.com pages. The distribution of the websites in Italian was very different from that in English. Government websites are by far the prevalent ones, with 42% (compared with 17% in English), followed by journalistic websites with 16%. Another striking difference in the search in Italian was that 3 commercial websites were present in the first 10 hits returned by Google, three times their frequency in the total of the 163 websites (*P* = 0.03 by one-tailed Chi-square test). Of these three websites, one was a company selling CAM products, supplements, and books on alternative medicine^3^; one was selling appointments with a naturopathic doctor, a “mini-market bio” and online sale of CAM herbs^4^; the third was a website from Vicks, a company selling over-the-counter products for cough and cold.^5^

**Figure 3:**
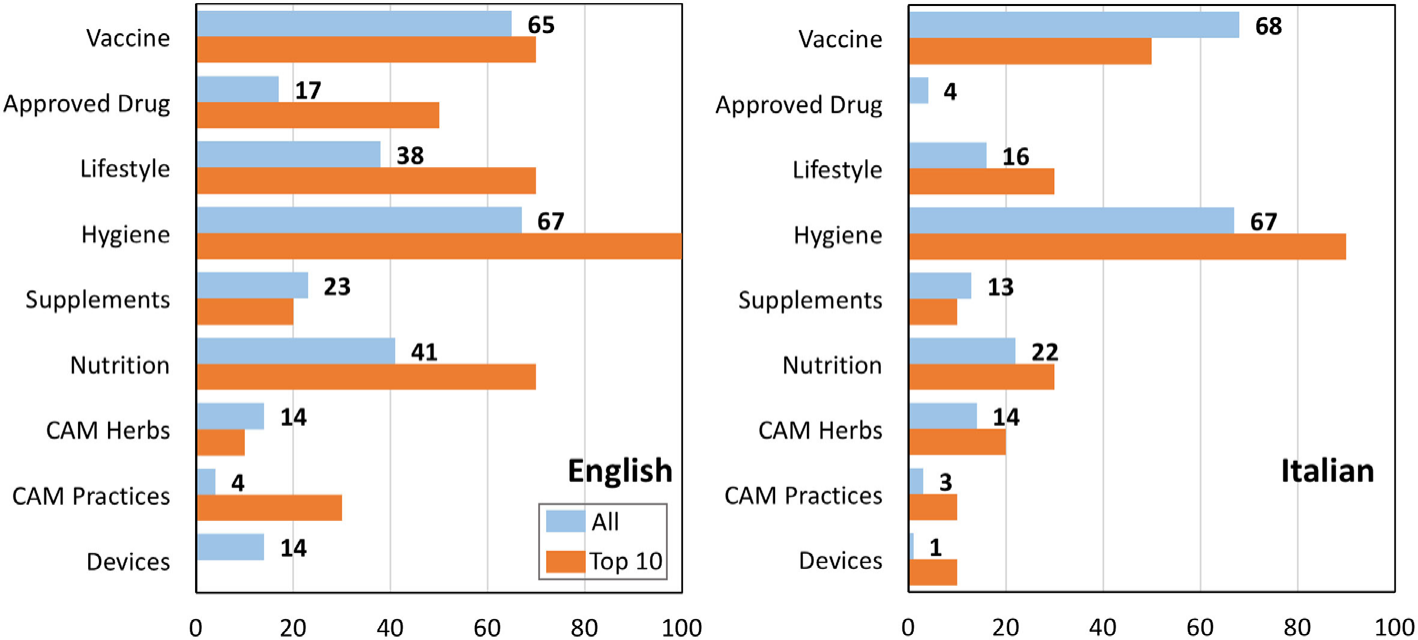
**Indication provided by various websites in a search on influenza prevention in English (left) or Italian (right)**. Data are expressed as the percentage of websites describing specific interventions (the total is >100% as many websites described more than one preventative approach). Blue bars, all websites; orange bars, top 10 results returned by Google.

An analysis of the indication provided by website on influenza prevention is shown in Figure 3. In both languages, the majority of the websites pointed at vaccination and hygiene (65–68%), two evidence-based approaches. Supplements or CAM herbs occurred less frequently, while two other non-evidence-based medicine (EBM) approaches, nutrition and lifestyle, were present in higher percentage in English when compared to Italian. There was no evidence for any of the prevention approaches being differentially represented in the top 10 searches.

**Figure 4:**
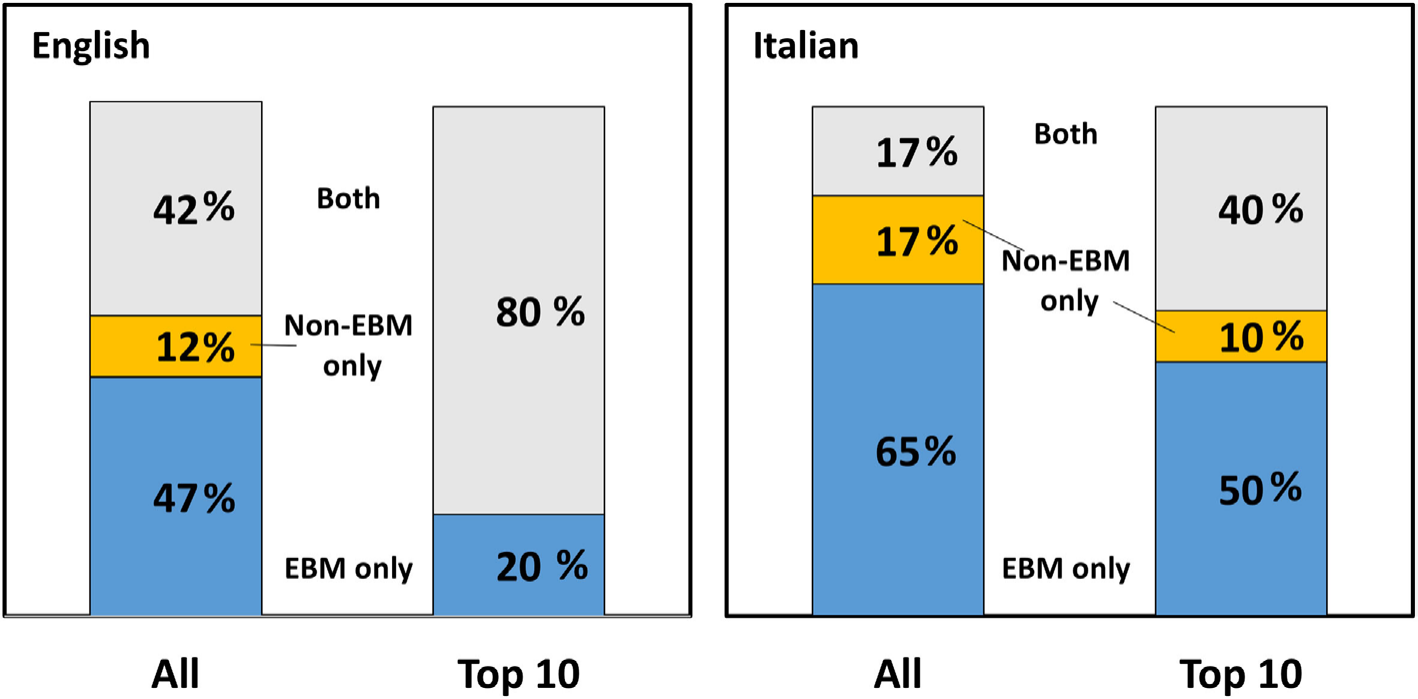
**Preventative approaches**. Percentage of websites on influenza prevention describing only EBM approaches (blue), only non-EBM (yellow) or both (gray) in English (left) or Italian (right). Both the whole search and the top 10 websites are shown.

We then attempted to analyze how many websites described only EBM-based preventative approaches (i.e., one or more of the following: vaccine, hygiene, approved drugs), only non-EBM (lifestyle, nutrition, CAM, supplements), or both. We did not include in this analysis the seven websites on devices as it was unclear which of these are EBM based. The results are shown in Figure 4. In the SERP in English, the majority of websites (almost 90%) described either only EBM, or both EBM and non-EBM approaches, and only 12% described only non-EBM ones. In the top 10 websites, 8 mentioned both EBM and non-EBM option and 2^6^ only EBM approaches. Therefore, Google ranking promoted the websites describing both EBM and non-EBM approaches, as these were 40% in the overall search and 80% in the top 10 (*P* < 0.05 by a two-tailed Chi-square test).

In a search in Italian, also shown in Figure 4, the overall pattern was similar except that the number of websites describing only EBM approaches was significantly higher than in English (101/163 in Italian vs. 85/185 in English, *P* = 0.002 by Chi-square test). Looking at the top 10 results in Italian, 4 mentioned both EBM and non-EBM option, 5 only EBM and 1 only non-EBM options. The most striking difference between Italian and English top websites was that the ones in Italian were less “neutral” (or more “partisan”) in that 60% (compared to 20% in English) only described one type of approach (EBM only or non-EBM only).

**Figure 5:**
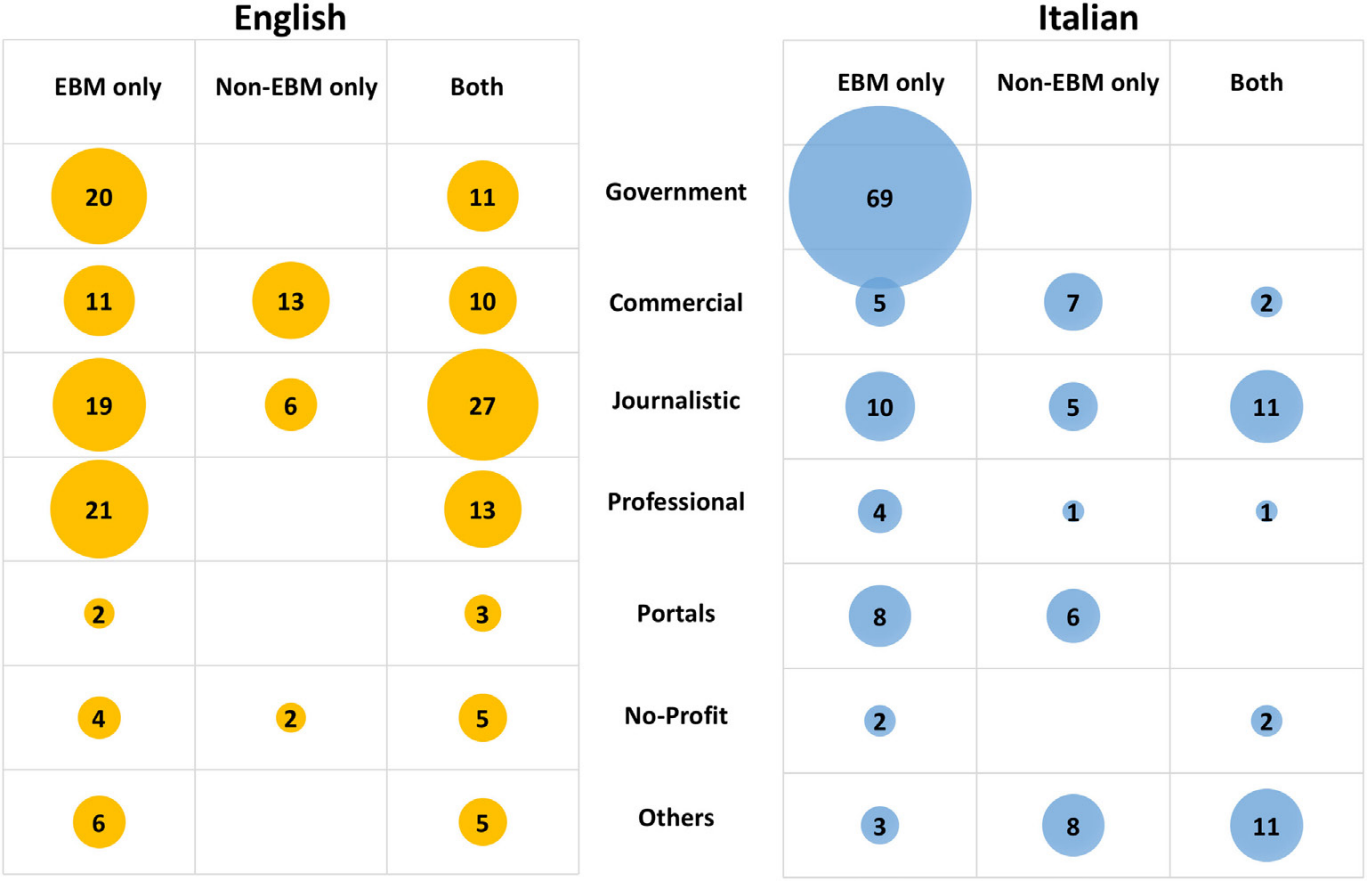
**Preventative approaches described by different types of websites**. Number of websites in different classes describing only EBM, only non-EBM, or both preventative approaches. Left panel, English; right panel, Italian.

These differences can, at least in part, be explained by an analysis of the typologies of websites in the three categories shown in Figure 5. In English (left panel), the most striking finding was that non-EBM-only websites did not have any government website but had a significantly higher proportion of commercial websites that represented 62% of this group, vs. 13 and 14% only in the other two groups. (*P* < 0.0001 vs. both groups by two-tailed Chi-square). In Italian (also in Figure 5) the large number of government websites accounts for the high percentage of websites reporting EBM-only approaches noted in Figure 4. A full breakdown of the different preventative approaches by class of websites is provided in Figures S1 and S2 in Supplementary Material for English and Italian, respectively.

When we analyzed the JAMA score of the websites in English, there was no difference among the three subgroups of types of intervention (mean ± SD was: EBM-only, 1.8 ± 1.0; non-EBM-only, 1.7 ± 0.9; both, 1.8 ± 1.0). However, in Italian, EBM-only websites had a significantly higher JAMA score (3.1 ± 1.2, *n* = 101) than websites mentioning non-EBM only (1.7 ± 1.1, *n* = 27) or both (1.8 ± 0.9, *n* = 35); *P* < 0.0001 comparing EBM only with any of the other two groups.

**Figure 6:**
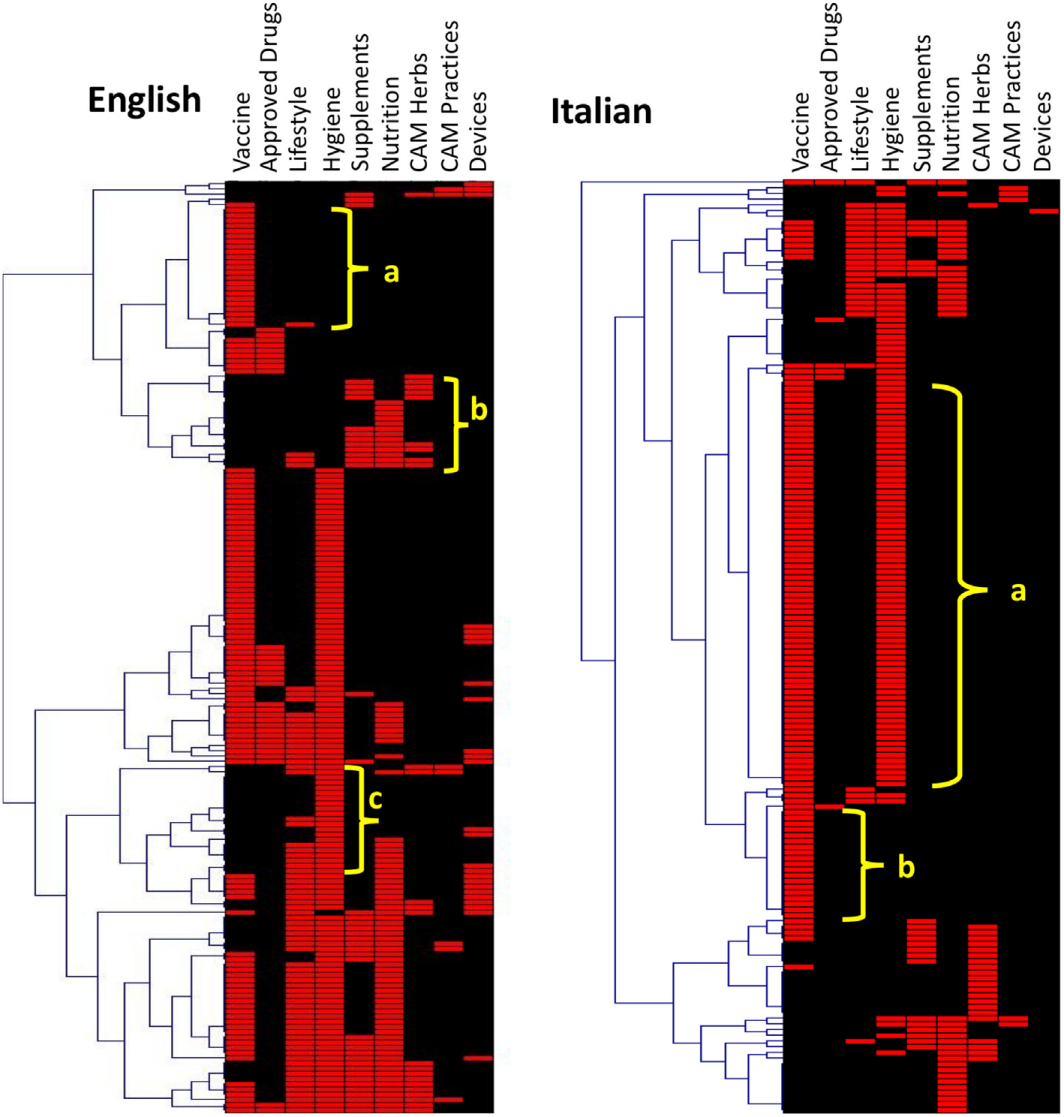
**Hierarchical cluster analysis of the websites on influenza prevention returned by Google**. Type of preventative intervention mentioned by websites on influenza prevention in English (left) or Italian (right). The clusters indicated are discussed in the text.

In order to be able to visualize patterns, we performed a cluster analysis of the various websites according to the preventative intervention they describe (Figure 6). In English (Figure 6, left), we evidenced four patterns. The first cluster (“a”) is made by 22 websites pointing exclusively at the vaccine. These were mainly journalistic sites (45%), followed by professional (18%), commercial (14%), government, and others (9%). Two clusters (“b” and “c”) are noticeable as they do not mention vaccines at all. In cluster “b,” of the 18 websites, 61% are commercial, and 33% journalistic. In this cluster, vaccination is not mentioned in favor of supplements, nutrition, and complementary medicine. This confirms the earlier conclusion that commercial websites are the less likely to point to vaccination as an option. Cluster “d” also does not recommend vaccines but favors mostly hygiene as a preventative approach. In this cluster, most of the 21 websites are also commercial (29%) followed by professional (22%), journalism (21%), and government (17%).

A cluster analysis of Italian websites on prevention (Figure 6, right) identified two major EBM-only clusters. The first (cluster “a”), recommending both the vaccine and hygiene measures, included 70 websites, of which 81% were government websites and none was a commercial website. Cluster “b” (vaccination only) had 19 websites of which 37% were government ones, confirming that these websites preferentially mention both main EBM preventative measures, hygiene, and vaccination.

### Searching Google for Influenza Vaccine

**Figure 7:**
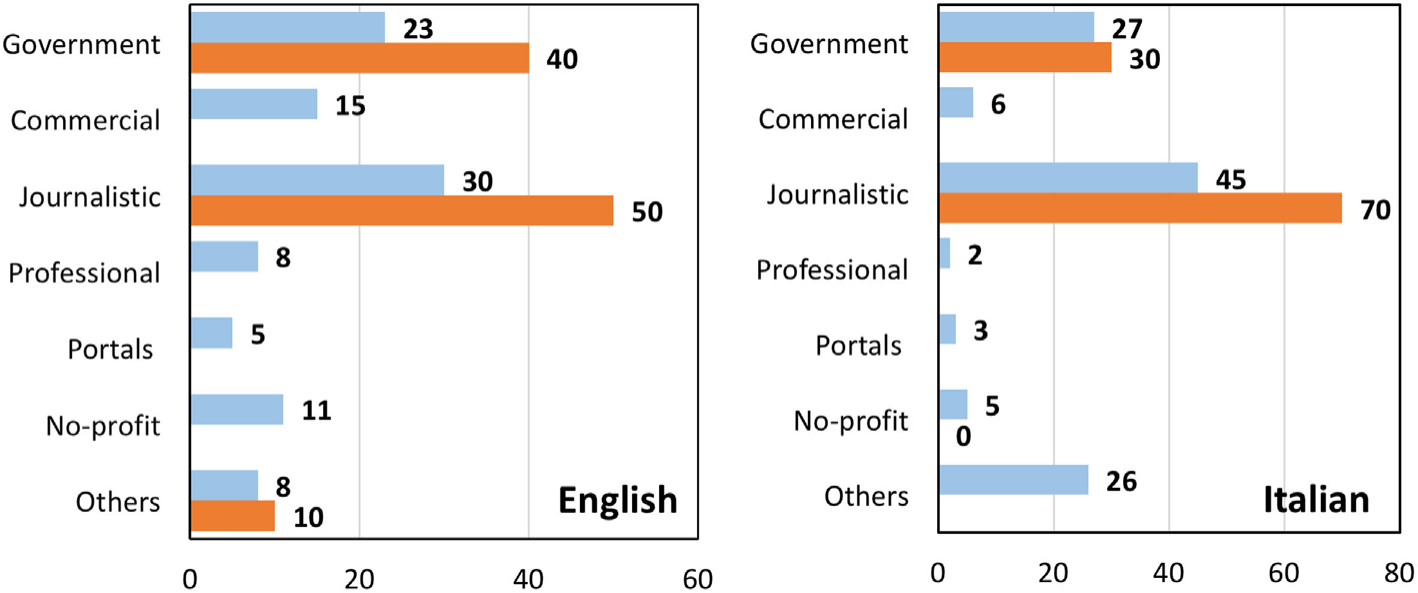
**Percentage of different classes of websites from a search on influenza vaccine in English (left) or Italian (right)**. Blue bars, all websites; orange bars, top 10 results returned by Google.

Figure 7 shows the types of websites returned by Google when searching for influenza vaccine in English or Italian. For each search, we analyzed the overall SERP (blue bars) or the top 10 websites only (orange bars). It can be seen that journalistic websites are the most numerous (30% in English, 45% in Italian) and have a high visibility in the top 10 results for both languages. Government websites follow with 23–27%.

**Figure 8:**
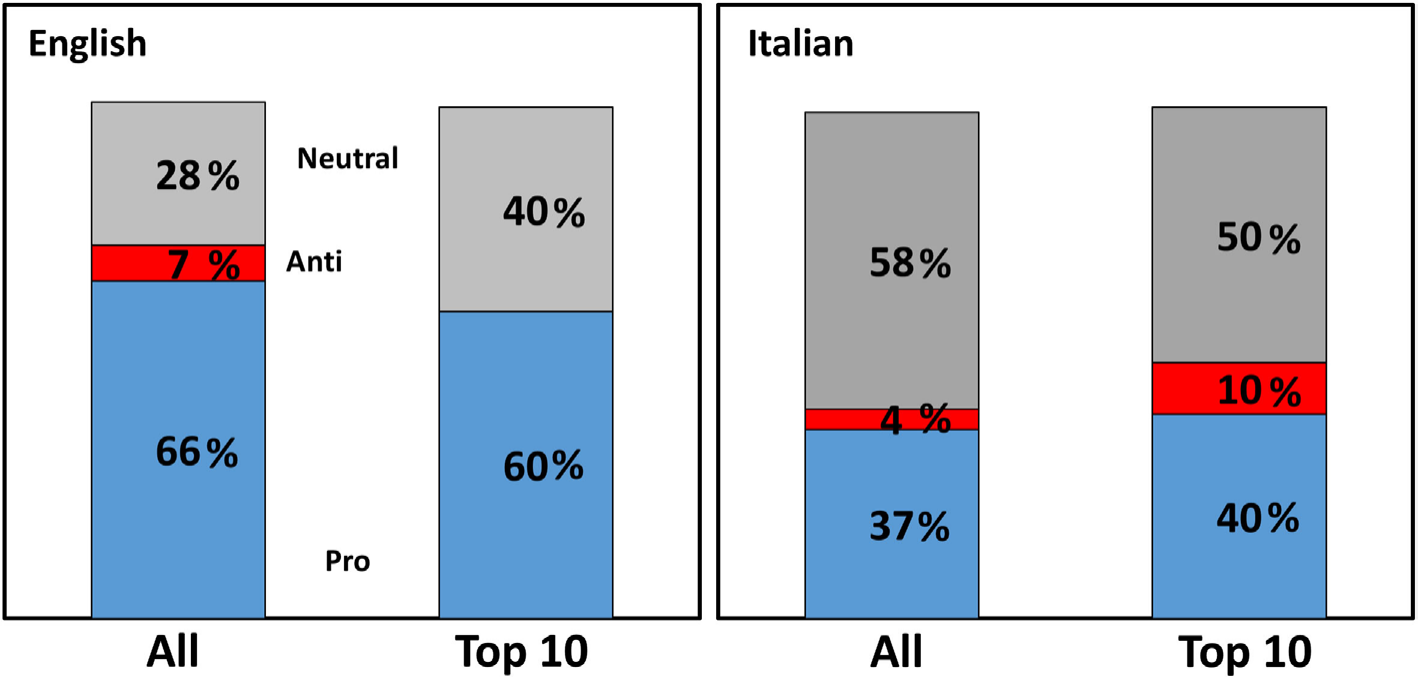
**Orientation of websites from a search on influenza vaccine**. Percentage of websites on influenza vaccine having a pro-vaccine (blue), anti-vaccine (red), or neutral (gray) orientation, in English (left) or Italian (right). Both the whole search and the top 10 websites are shown.

**Figure 9:**
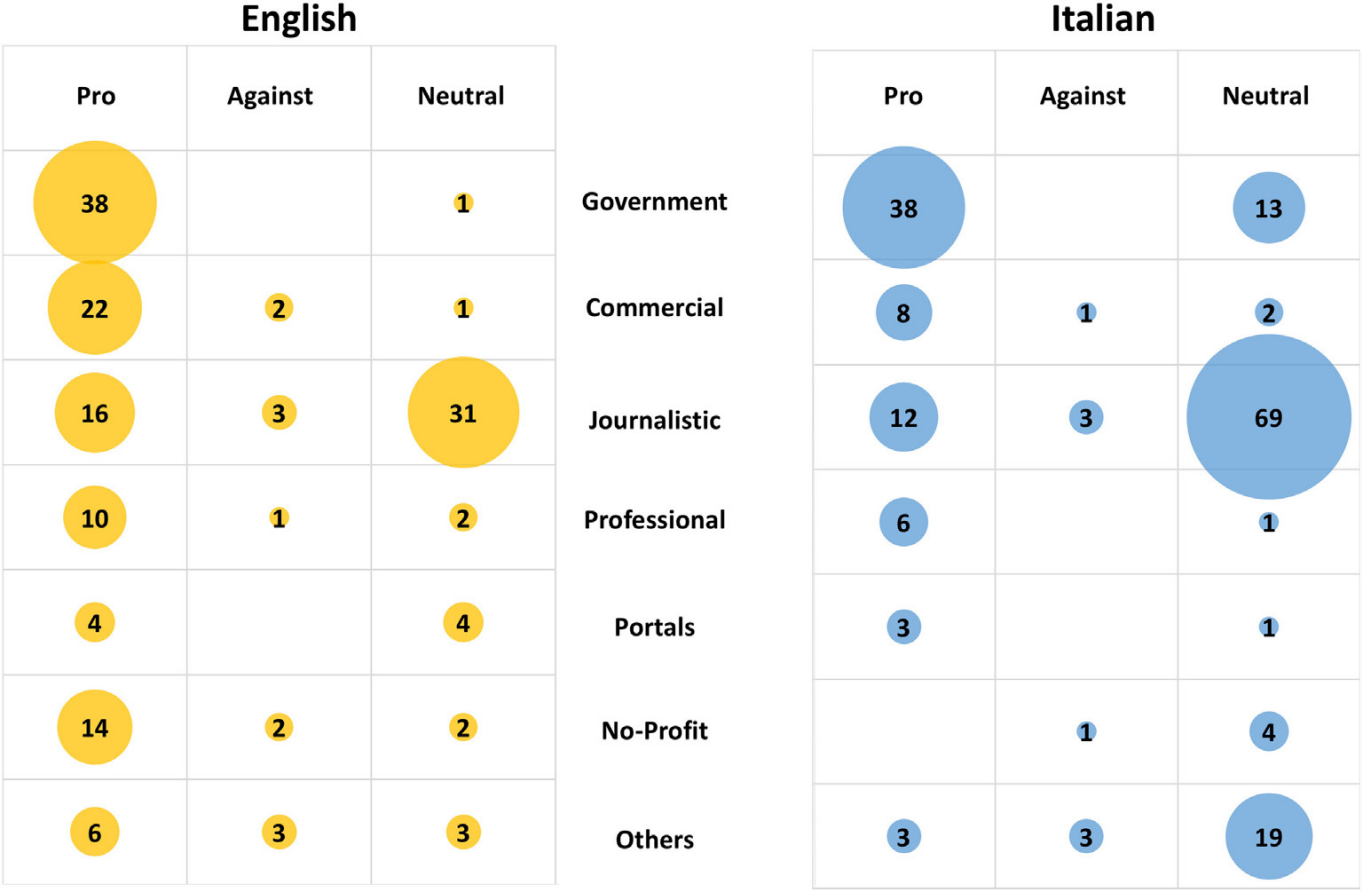
**Orientation on influenza vaccine by different types of websites**. Number of websites in different classes having a pro-vaccine, anti-vaccine, or neutral orientation. Left panel, English; right panel, Italian.

When these websites were analyzed for the recommendations made, either pro-vaccine, anti-vaccine, or neutral, the majority of websites in both languages were pro-vaccination or gave a neutral information, the latter prevailing in Italian websites (Figure 8). A similar distribution was observed in the top 10 results with the exception that, in Italian, an anti-vaccine website made it to the top 10. This was an article by a vaccine-skeptic doctor published in the online comments section of the newspaper “il fatto quotidiano.”^7^ As shown in Figure 9, a government affiliation was associated mainly with pro-vaccine statements, with a few giving neutral information (such as opening hour of a surgery). Although there were few anti-vaccine websites in English, we can notice a significantly higher (*P* = 0.04) proportion of “other” websites (that include blogs and personal websites) (25% of the anti-vaccine group vs. 8% in the overall SERP). The other striking observation, more evident in Italian, was the high proportion of journalistic websites providing information that we classified as “neutral.”

While the proportion of journalistic websites was the same in the two searches in English (30%, both for prevention and the vaccine search – Figures 1 and 7), in Italian, they were significantly (*P* < 0.0001) more frequent in the search of the vaccine (45%) than in that on prevention (16%).

When we analyzed the JAMA score from the websites returned from the vaccine search according to class of websites, there was a trend, in both languages, for anti-vaccine websites to score higher than pro-vaccine websites, but this was not statistically significant (English JAMA scores were: pro-vaccine, 2.3 ± 1.3; anti-vaccine, 3.1 ± 1.2; neutral, 2.8 ± 1.3. Italian JAMA scores were: pro-vaccine 2.2 ± 1.1, anti-vaccine, 2.5 ± 1.4; neutral, 2.2 ± 0.8). When the JAMA score for all four searches was analyzed by class of websites (Tables S5 and S6 in Supplementary Material), in both languages, commercial websites had a significantly lower JAMA score, a pattern that had been observed in previous studies (22, 25). Journalism websites, on the contrary, had a significantly higher score, which was also found previously (22).

### Impact of News on the Websites Returned by Google

Because the results reported above show a large proportion of journalistic websites and their high ranking in a Google search on influenza vaccine, we used a NLP technique to analyze the two corpora on journalistic websites returned in both languages. In English, a corpus analysis of the 50 journalistic websites revealed 38 occurrences of the string “Public Health England” and 15 of “Center for Disease Control” (full frequency list of tri-grams is shown in Data Sheet S2 in Supplementary Material, sheet 1). A similar search in the whole corpus represented by the search on “flu jab” revealed many more occurrences of these strings. Analyzing the context where this expression was used (full concordance for the string “Public Health England is shown in Data Sheet S2 in Supplementary Material, sheet 2) and further manual scrutiny revealed many websites were reporting the same news, namely, the very low efficacy of the vaccine in the 2014/2015 season. Thirteen were based on a press release from Public Health England reporting that the vaccine used in the UK was only 3% effective (5–6 January 2015). Similar news was published in various English speaking countries. Four (5–10 December 2014, one journalistic website, two portals, one blog an anti-vaccine no profit website) were based on a news release from the Center for Disease Control reporting a low efficacy of the vaccine. Five such journalistic websites were in the top 10 results of the Google SERP.

We then analyzed the 83 journalistic websites from the search on the vaccine in Italian. Calculating the ngram frequencies in this corpus, and eliminating common words and all those derived from the words vaccine, vaccination, and influenza, the second bi-gram in the list was “morti sospette” (suspicious deaths), occurring 78 times in this corpus (full word list of bi-grams is shown in Data Sheet S2 in Supplementary Material, sheet 3). Analyzing the context where this expression was used (see the concordance in supplementary material; Data Sheet S2 in Supplementary Material, sheet 4), it was clear that this referred to a series of news articles reporting the news that the influenza vaccine could have caused the death of up to 13 elderly people, a connection then ruled out by the Italian and European public health authorities. Two such news websites were in the top 10 websites, one from the magazine “Altroconsumo” and one from the financial newspaper “il Sole 24 Ore.”^8^

## Discussion

### Typology of Websites

The present study analyzed four SERPs with 200 websites, each on the prevention of influenza or its vaccine. The relatively large number of websites provides a good sample for the analysis of the composition of the SERP in terms of typology of websites and their orientation. In addition, we looked at the composition of the first ten websites and compared them with that of the whole 200-websites SERP to gain information on the visibility of websites. We are aware that this is a sort of reverse engineered search engine optimization, because the algorithm used by Google is not public. However, website visibility is important because users, in most cases, do not go beyond the first results in the SERP (14, 15).

We found that, at least in English, contrary to a common prejudice that Google might privilege market-oriented or promotional sources, commercial websites are ranking low and are not present in the top 10 websites in either the SERP on influenza prevention or that on the vaccine. This is in agreement with findings from our previous studies on IQ on two Google searches on diabetic neuropathy or migraine where commercial websites, that accounted for 20 or 30% of the 200-website SERP, respectively, were absent in the top 10 results (22, 25). The low ranking of commercial websites is aligned with users’ behavior as studies on trust in online advice have shown that, both in financial or health matters, trust is negatively influenced by the perception of commercial intentions (30, 31). This was not true in Italian where three commercial websites were in the top 10.

### Orientation of Websites

Another concern often voiced in studies on health IQ is that search engines could give high visibility to non-scientific websites or point users to low-quality information. Google ranks websites by multiple criteria and it is not clear how much of the content is taken into account. We used the type of intervention suggested as an indicator as to whether the information provided was in accordance with the principles of EBM. For this, we relied on the conservative indications provided by the Cochrane collaboration or the UK National Institute for Health and Care Excellence. We are aware of the potential problems and the fact that this could underestimate the potential of some treatments, but these organizations are often seen as setting the “gold standard.”

Google gives a fair balance of websites favoring EBM-informed approaches. This is particularly evident in English where the non-EBM-only prevention website ranking highest was 39^th^, although from a different perspective, the fact that 80% of the top 10 websites described both EBM- and non-EBM approaches, which could be a problem if people thought it would be easier to eat tomatoes rather than taking the vaccine or washing their hands. In Italian, the trend was similar with 17% of websites pointing to non-EBM-only preventative approaches, but here the first such websites ranked 6th and the next 12^th^, and thus had better visibility.

Likewise, in English, anti-vaccination websites do not show in the first 10 results, the first anti-vaccine website ranking 62nd. However, in Italian, although there were less anti-vaccine websites, one such website was 1st in the ranking and the next one 33rd.

**Figure 10:**
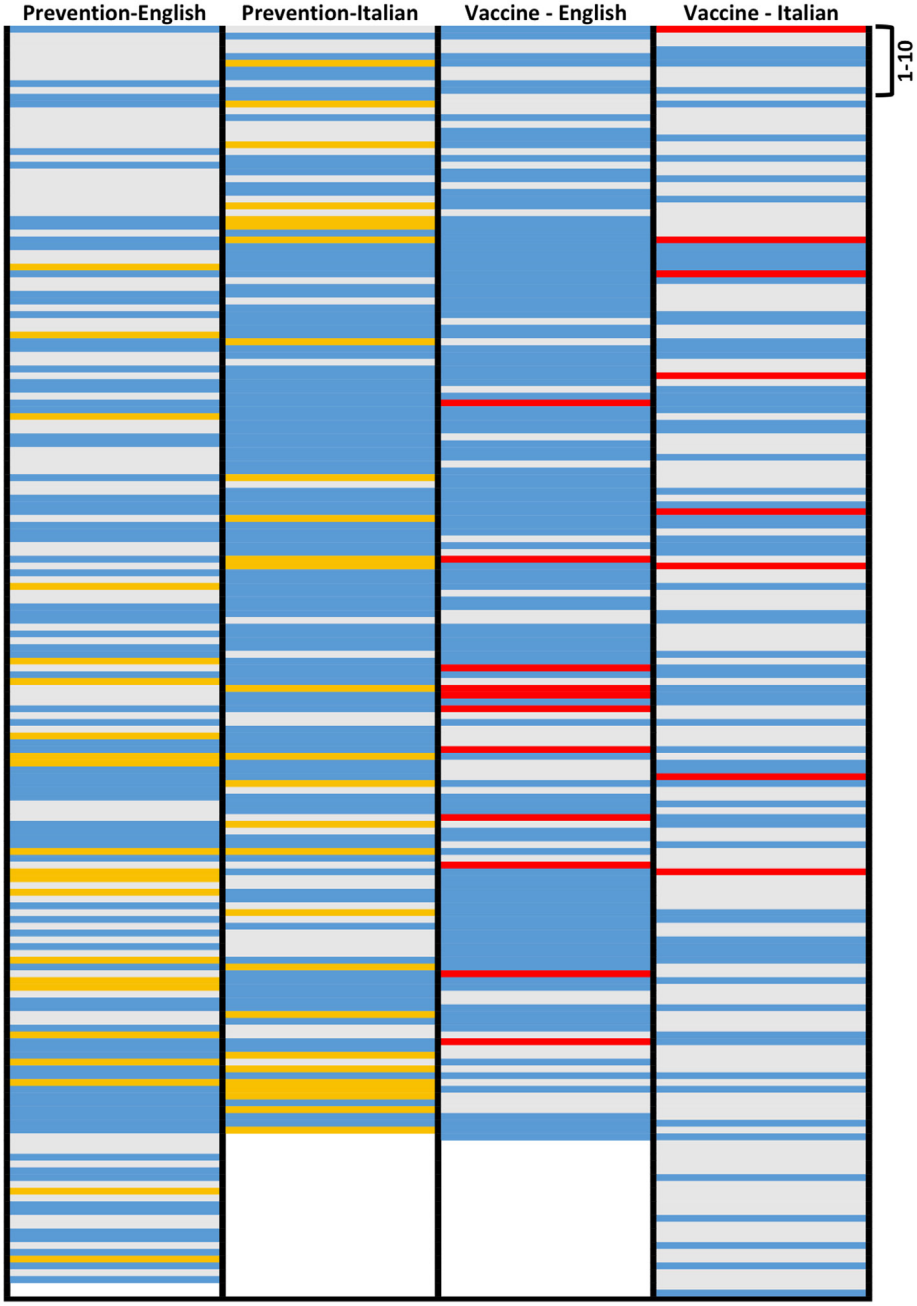
**Graphical representation of the ranking of websites with different orientation in English and Italian**. Left two columns (search on influenza prevention): only EBM approaches, blue; only non-EBM, yellow; both, gray. Right columns (search on vaccine): pro-vaccine, blue; anti-vaccine, red; neutral, gray.

In general, in Italian, non-EBM-only websites and anti-vaccine websites are more likely to rank higher than in a search in English, as summarized in Figure 10. The reason for this is not clear because the Google ranking is based on an automated evaluation of IQ by some intrinsic characteristics of websites, without an assessment of its content, such as readability, structure, links, and the same considerations we made above on commercial websites in Italian apply.

It should also be noted that our observation that anti-vaccine websites in English are ranked low by Google is in contrast with previous reports that 2–10 of the top ten websites resulting from a search on “vaccination” or ”immunization” were anti-vaccine websites (32, 33). One explanation could be that those studies were on searches on vaccination in general, and most of the anti-vaccine websites focus on those vaccines, such as the MMR vaccine, which are made compulsory in many countries, while the influenza vaccine is only recommended to populations at risk, and therefore, raises less opposition.

It is also interesting that commercial websites are frequently recommending non-EBM treatments, but this was probably to be expected as pharmaceutical companies do not sell or advertise their drugs on the internet but rely on prescriptions or doctors’ recommendation. Likewise, the generally pro-vaccine information of government websites is not surprising as many of these websites were in fact set up to promote a vaccination campaign. This seems to be an effective measure as a Canadian study on 250 pregnant women found that those who relied on government agencies’ websites were more likely to be vaccinated than those relying on websites from mainstream media (34).

### Indicators of Trustworthiness

The analysis of the JAMA score indicated that this is not predictive of whether a website is describing EBP-approaches or not (except in Italian), or whether they are pro- or anti-vaccine. On the other hand, we confirmed our earlier observations that commercial websites have a lower JAMA score (22, 25). It is interesting to hypothesize that the lack of some quality indicators of a document, such as date, author, references – all components of the JAMA score, may contribute to the low ranking of commercial websites by Google.

Another established criterion for health IQ is having the website approved by the Health-On-The-Net foundation and displaying the HON seal of approval. However, crawling the websites of our four searches using NLP software we searched for text references to the HON seal, and we only found 4 websites containing text references to HONcode (one for each of the four SERPs) l.^9^ Probably the fact that the HON seal is something that a webmasters need to apply for and has a cost, which is not clarified on the HON website, may limit its diffusion. In addition, at list in our search, it is not displayed by websites from government agencies, public healthcare services or Universities.

### News

The impact on news websites deserves more discussion. First of all, they give a more “balanced” view and this is the class of websites that is more likely to describe both EBM and non-EBM approaches, in both languages (Figure 5) and to describe the influenza vaccine in a “neutral” way (with the caveat discussed below). This has also been noted in a Dutch qualitative study similarly reported that news websites are more objective and non-judgmental while a critical view of vaccination was more common in social media (35). On the other hand, journalism websites are not very well represented among those recommending the vaccine when searching for preventative strategies.

The textual analysis of the journalistic websites shows that, for the past winter, they all gave the same type of news, different in the two languages. This is probably due to the fact that newspapers are nowadays understaffed and tend to just re-post press releases rather than having articles written by their own scientific journalists. English websites mainly reported data from government agencies describing the low efficacy of that year’s vaccine. Although most newspapers reminded the reader that, despite the concerns reported, the government still recommended vaccination for those at risk, the titles had a negative tone (Sky News: “flu jab found to work in just 3% of cases”^10^; Daily Mail, “Flu jab is a waste of time for 97% of patients”^11^).

The news websites in Italian were even more peculiar. On 28th of November, 2014, all main newspapers published in the front-page headline that three elderly people died within 2 days from having been vaccinated (e.g., il Corriere della Sera and il Giornale^12^) and panic ensued. The health authorities suspended two batches of vaccine and the number of “suspicious deaths” reached 13, as reported in the front page of il Sole 24 Ore.^13^ The emergency did not last long and a week later newspapers reported (with much less emphasis) that the health authorities have concluded that those deaths were not associated with the vaccine, which was confirmed on 3 December 2014 by the Pharmacovigilance Risk Assessment Committee of the European Medicines Agency (EMA).^14^

As early as the 29th of November, Dr. Cirielli, president of the Società Italiana di Medicina Generale (Simg) noted that every day in Italy 1,600 over-65s die; as the uptake rate for the influenza vaccine in that population is over 50%, 400 people die every day “after having been vaccinated.”^15^ Thus, it would require a highly powered study to attribute three deaths to the vaccination.

It is nuclear what have sensitized the public opinion to report those deaths. One interesting coincidence is that three days earlier, the 25th of November, most newspapers reported that judge Di Leo of the Tribunale del Lavoro di Milano (employment tribunal), based on an 18-page report from forensic doctor Alberto Tornatore, concluded that a hexavalent vaccine (against, among others, *Haemophilus influenzae*) had probably caused autism in a child and sentenced the Italian government to pay monthly compensation.^16^

Thus, in each language, a language-specific “bad news” made up a very significant portion of the journalistic websites on the influenza vaccine. There was a small overlap between the two SERP on vaccine in that two Italian websites reported the data of low efficacy released by the CDC^17^ and two websites in English reported the news of the suspected accident in Italy.^18^

Many studies have investigated the quality of health information available on the web, often with a preconception that either the uncontrolled nature of the web will allow posting of low quality information, misinformation or disinformation. In this perspective, the layperson not being able to discriminate between low-quality and high-quality sites, there is a concern that the use of the Internet by the patient may cause harm by promoting potentially unsafe treatments (36), although others found little evidence of this in the medical literature (37).

## Conclusion and Limitations

In drawing conclusions, one should be aware of the limitation of this study. Search results vary with time; different search terms or health topics will give different results; searching from a different location (as defined by the IP address) will give different results. Although some findings were in agreement with previous studies from our group performed in the last three years across different searches (such as commercial websites ranking lower and having a lower JAMA score), other findings may not be generalized.

The other finding that would be worth confirming further is that most websites returned are not “standalone websites” but are in fact documents and publications from professional organizations, newspapers, government agencies, and commercial companies. If this was generalized, then the idea is that the Internet is a source of uncontrolled information where anyone can make any statement.

Another important finding was that, for relatively hot topics, Google returns a high proportion of journalistic websites from TV channels or print journals. This raises the interesting theoretical point on whether “newsworthiness” should be considered an indicator of IQ like trustworthiness or if it is a confounder, as well as the role of journalist as the gatekeeper. When we discuss health IQ, we think of information as “knowledge obtained from investigation, study, or instruction” but the word also defines “intelligence or news” (Merriam-Webster Online Dictionary). It is not clear whether the ways we assess IQ are the same for the two definitions of “information.” Also, we do not know which ones of the top 10 websites a user will actually read and whether they will give the same weight to an article in the press or a different type of information. This would be an important follow up of this study.

It would be important to know whether the different pattern in Italian vs. English is observed with other search terms, and to what extent this happens with other non-English languages. It is also curious that only “bad news” received so much attention. An earlier study on news on medical research in the press had concluded that “Newspapers underreported randomized trials, emphasized bad news from observational studies, and ignored research from developing countries” (38).

**Figure 11:**
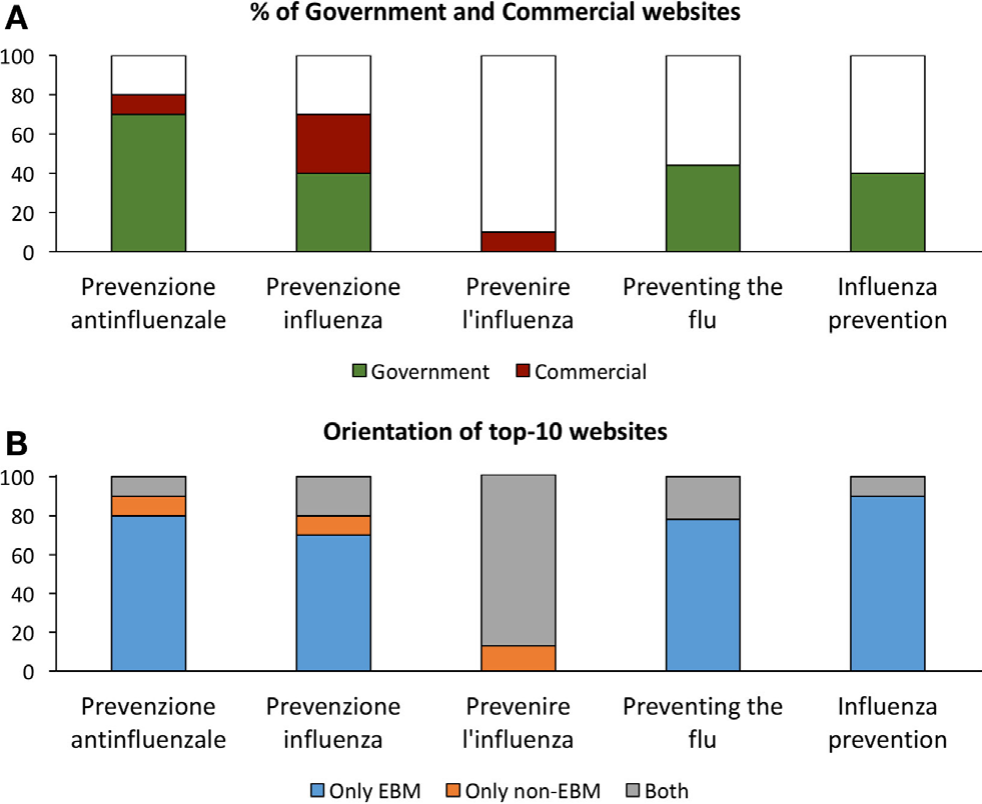
**Percentage of government and commercial websites and their orientation in the top 10 results searching different terms**. Searches for the indicated terms, the first three in Italian, the last two in English, were performed on 15/11/2015 and the first 10 fits analyzed. **(A)** Percentage of commercial or government websites. **(B)** Percentage of websites on prevention describing only EBM approaches (blue), only non-EBM (yellow), or both (gray) in Italian (left) or English (right).

As suggested in the peer review process, we performed a search on the prevention of influenza comparing different search terms. In English, we searched for “preventing the flu” and “influenza prevention”; in Italian for “prevenire l’influenza,” “prevenzione influenza” and “prevenzione antinfluenzale” and analyzed the top 10 websites. We investigated: (1) the percentage of commercial websites and (2) the orientation (EBM vs. non-EBM). The results are shown in Figure 11 (raw data are available in the Supplementary Material as Data Sheet S3 in Supplementary Material). Because this search was performed several months later, it cannot be compared to the ones above. However, the results confirm the main messages of the study, that is, that a search in Italian gives more visibility to commercial websites and to non-EBM websites than those in English. In fact, a search in Italian for “prevenire l’influenza” (which is a less formal language than without the other search terms without the article) returns no government websites and is more oriented toward non-EBM terminology as compared to the results with other search terms. This probably should suggest government agencies to ensure that informal language as it may well be that official government in Italian are written in a more technical language than their correspondent texts in English and should carefully consider this point when performing search engine optimization. The results also suggest performing future research on the impact of the language style on the quality of health information returned by Google.

Finally, it is important to monitor the impact of web health information on public health. The output of search engines can affect knowledge and attitude about vaccination significantly, (39) and thus might impact on the uptake of vaccination (40). It would be interesting to have a follow-up epidemiological study to assess how the health information available in the press has influenced the vaccine uptake in the next year.

## Author Contributions

PG, AM: designed research, analysed the data, and wrote paper. RE: designed research, wrote paper.

## Conflict of Interest Statement

The authors declare that the research was conducted in the absence of any commercial or financial relationships that could be construed as a potential conflict of interest.

## Footnotes

^1^www.google.com

^2^www.sketchengine.co.uk

^3^http://www.guna.it/prevenzione-invernale (archived in: http://www.webcitation.org/6Z9XJAxUH)

^4^http://www.lucaavoledo.it/2011/10/prevenzione-naturale-di-influenza.html (archived in: http://www.webcitation.org/6Z9Xgb9Mr)

^5^http://www.vicks.it/saperne-di-piu/articoli-prevenzione/articoli/raffreddore-influenza-bambini/ (archived in: http://www.webcitation.org/6Z9a82Vfi)

^6^www.cdc.gov and www.mayoclinic.org

^7^http://www.ilfattoquotidiano.it/2014/12/09/vaccino-antinfluenzale-dati-mortalita-fede/1258733/ (archived in: http://www.webcitation.org/6Z8VM2Ipp)

^8^http://www.altroconsumo.it/salute/influenza/news/vaccino-antinfluenzale-ritirato-fluad-novartis (archived in: http://www.webcitation.org/6Z8YgS4gz); http://www.ilsole24ore.com/art/notizie/2014-11-29/vaccini-antinfluenzali-morti-sospette-salgono-12-ministro-lorenzin-primi-test-negativi-220813.shtml?uuid=ABiFgtJC (archived in: http://www.webcitation.org/6bjY7VCjs).

^9^Prevention in English, http://www.everydayhealth.com/cold-and-flu-pictures/ways-to-prevent-colds-and-flu.aspx; prevention in Italian, http://www.abcsalute.it/blog/influenza-americana-inverno-2014-cose-e-come-curarla/; vaccine in English, http://www.everydayhealth.com/drugs/influenza-virus-vaccine-live-trivalent-http://www.webcitation.org/6buN57wQn; vaccine in Italian, http://www.farmacoecura.it/influenza/influenza-2008-2009-vaccino-prevenzione/)

^10^http://news.sky.com/story/1422308/flu-jab-found-to-work-in-just-3-percent-of-cases (archived in: http://www.webcitation.org/6bvdaI4M9)

^11^http://www.dailymail.co.uk/news/article-2941896/Flu-jab-waste-time-97-patients-Vaccine-developed-year-ago-no-longer-matches-virus-mutated-much.html (archived in: http://www.webcitation.org/6Z9hZ28b2

^12^http://archiviostorico.corriere.it/2014/novembre/28/Vaccini_morti_sospette_caos_co_0_20141128_e7fda2a2-76d2-11e4-b904-b99b9498524a.shtml (archived in: http://www.webcitation.org/6bsuHeVY2); http://www.ilgiornale.it/news/politica/serie-morti-vaccino-panico-regioni-nel-caos-1071444.html (archived in: http://www.webcitation.org/6bsugIYSo)

^13^http://www.ilsole24ore.com/art/notizie/2014-11-29/vaccini-antinfluenzali-morti-sospette-salgono-12-ministro-lorenzin-primi-test-negativi-220813.shtml?uuid=AbiFgtJC (archived in: http://www.webcitation.org/6bsusggIm)

^14^http://www.ema.europa.eu/ema/index.jsp?curl=pages/news_and_events/news/2014/12/news_detail_002228.jsp&mid=WC0b01ac058004d5c1; http://www.webcitation.org/6bu31e9mg (archived in: http://www.webcitation.org/6bvfp65Vq)

^15^http://www.quotidianosanita.it/scienza-e-farmaci/articolo.php?articolo_id=24648; archived in: http://www.webcitation.org/6bu3d4t4R

^16^http://www.repubblica.it/salute/medicina/2014/11/25/news/il_tribunale_bimbo_autistico_per_colpa_del_vaccino-101357013/ (archived in: http://www.webcitation.org/6bswRLubo)

^17^http://www.assis.it/cdc-questanno-il-vaccino-antinfluenzale-e-pressoche-inefficace/ (archived in: http://www.webcitation.org/6bssq8yTV); http://www.terranuova.it/Medicina-Naturale/Vaccino-antinfluenzale-ora-si-scopre-che-e-anche-inefficace (archived in: http://www.webcitation.org/6bstEgxJV)

^18^http://time.com/3610872/italy-flu-vaccine-deaths/ (archived in: http://www.webcitation.org/6bstODqvC); http://blogs.wsj.com/pharmalot/2014/12/01/italy-investigates-novartis-flu-vaccine-after-12-deaths-are-reported/ (archived in: http://www.webcitation.org/6bstTpbD1)
