# Supplementary figures and images for: Bad News: Analysis of the Quality of Information on Influenza Prevention Returned by Google in English and Italian
Source: Front Immunol. 2015 Dec 8;6:616. doi: 10.3389/fimmu.2015.00616 (PMC4672033; doi:10.3389/fimmu.2015.00616)

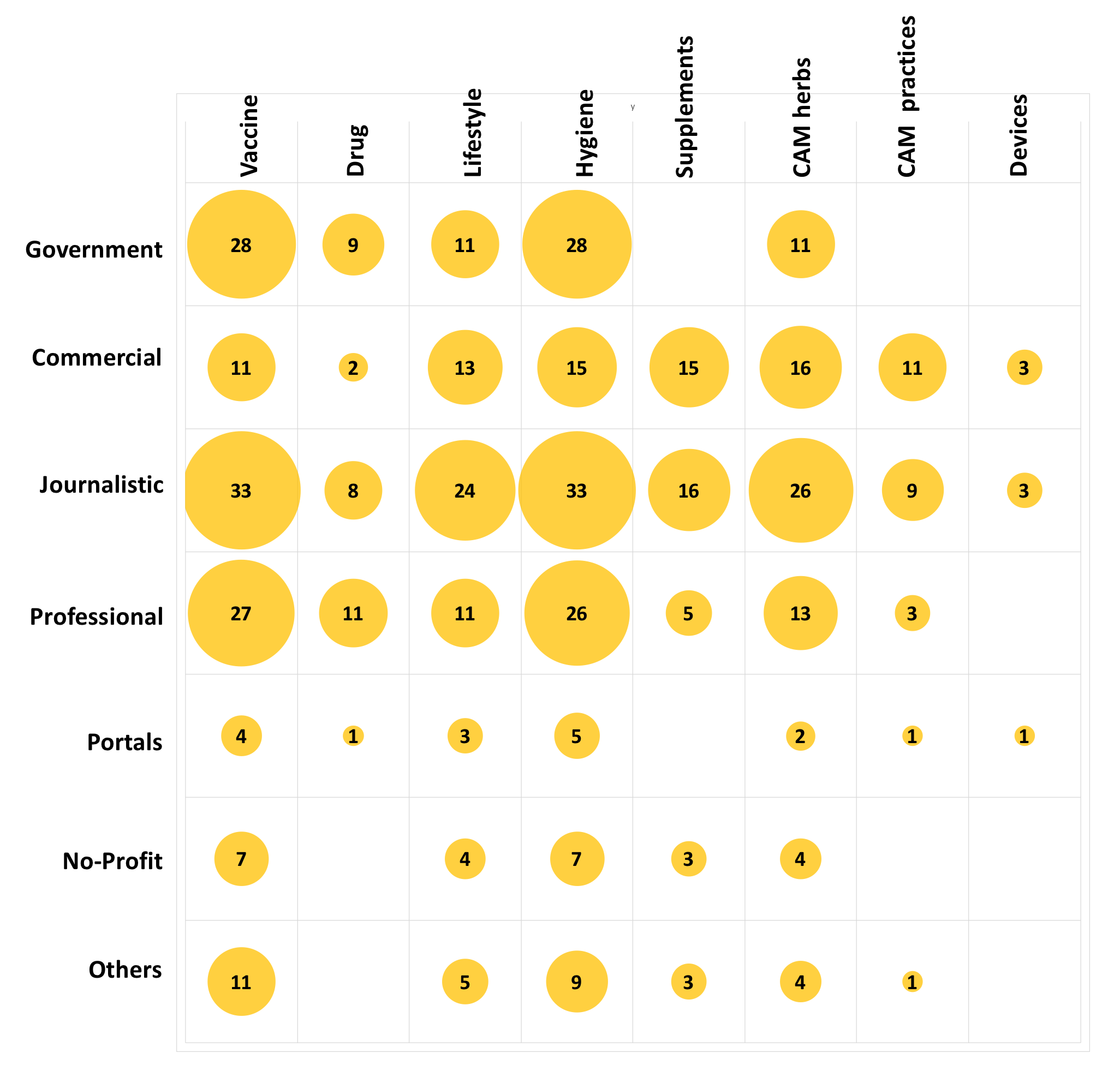

Supplement: Supplementary file 4 [file Image_1.TIF]

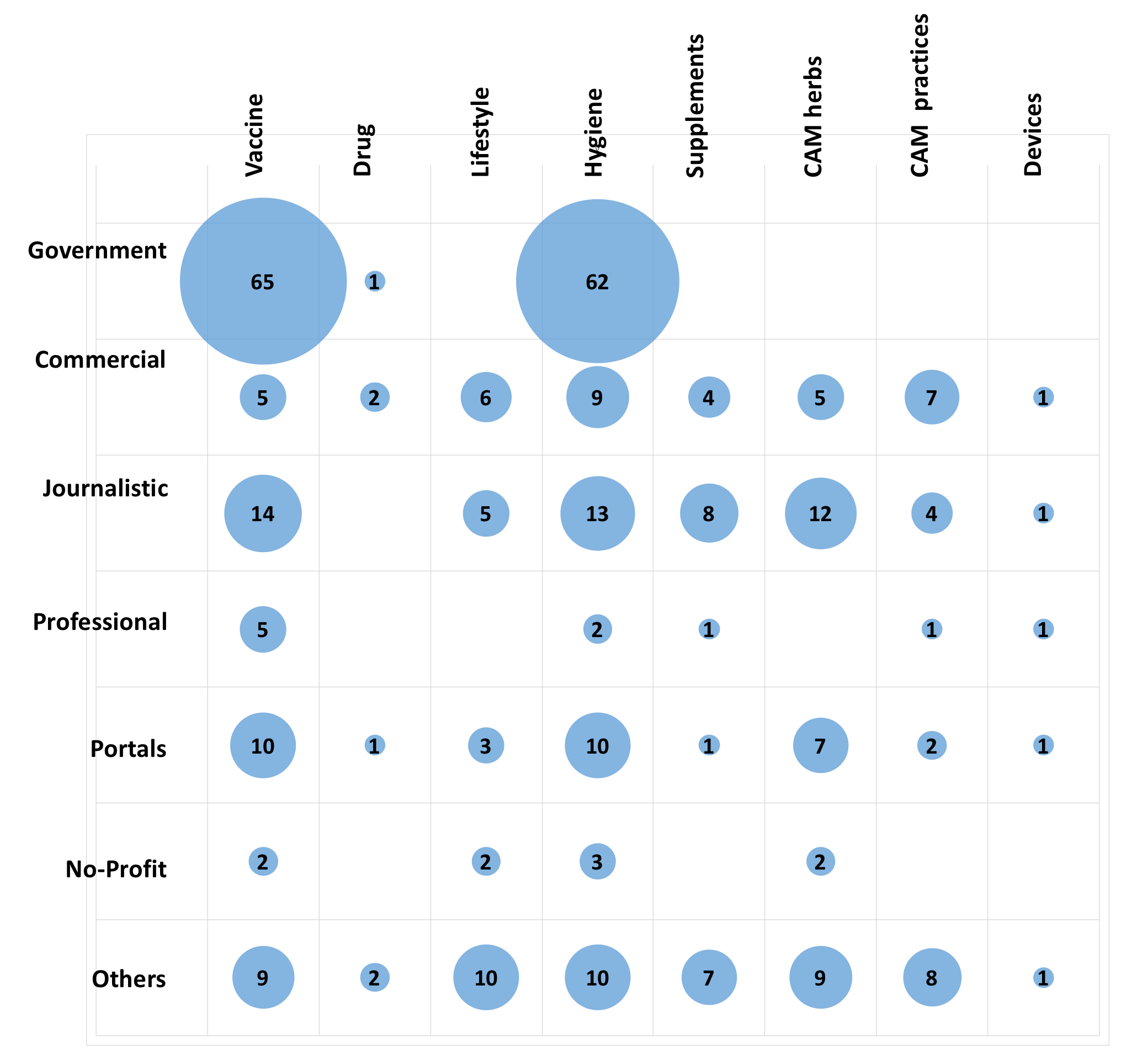

Supplement: Supplementary file 5 [file Image_2.TIF]
